# Assessment of patient satisfaction towards auditable pharmaceutical transactions and services implemented in outpatient hospital pharmacy in Ethiopia

**DOI:** 10.1186/s40545-021-00372-1

**Published:** 2021-10-19

**Authors:** Ayalew Adinew, Mamo Feyissa, Berhanu Tadesse, Birhanu Demeke, Tamrat Assefa, Mahdi Abdella, Edessa Diriba, Regasa Bayisa, Elias Geremew, Fikresilasie Alemu, Edmealem Ejigu, Tesfaye Seifu, Aschalew Nardos, Demelash Dejene, Mekete Mideksa, Natnael Solomon

**Affiliations:** 1 USAID Global Health Supply Chain Program-Procurement and Supply Management (GHSC-PSM)-Ethiopia, Addis Ababa, Ethiopia; 2 Department of Pharmacology and Clinical Pharmacy, School of Pharmacy, College of Health Sciences, Addis Ababa University, P.O. Box: 9086, Addis Ababa, Ethiopia; 3 Pharmaceutical and Medical Equipment Directorate, Federal Ministry of Health-Ethiopia (PMED/FMoH), Addis Ababa, Ethiopia; 4 School of Pharmacy, College of Health Sciences, Wollo University, Dessie, Ethiopia; 5 School of Pharmacy, College of Health Sciences, Awassa University, Awassa, Ethiopia; 6 Sidama Regional State, Health Bureau, Awassa, Ethiopia

**Keywords:** Pharmacy services, Hospital pharmacy, Patient’s satisfaction, APTS

## Abstract

**Background:**

Patient satisfaction is a widely used indicator to measure quality of pharmacy services. Currently, a transformational pharmacy service called auditable pharmaceutical transactions and services is being implemented nationally in Ethiopia. However, there is a dearth of evidence regarding the national impact of this system on patient satisfaction.

**Objective:**

To assess patient satisfaction in hospital pharmacies that have implemented auditable pharmaceutical transactions and services in Ethiopia.

**Method:**

This is a national study conducted based on a cross-sectional study design. Data were collected using a structured questionnaire from September 5 to October 5, 2020. The collected data was analyzed using spreadsheet excel and Statistical Package for the Social Sciences (SPSS) version 23. The proportions, ratios, and percentages were used for presenting data. A binary logistic regression test was used to determine the association of patient satisfaction with dispensary infrastructure, medicines availability, scores of labeling, and scores of patient knowledge on dispensed medicines. A *p* value < 0.05 was considered statistically significant.

**Result:**

A total of 650 participants were included in this study for whom a total of 1422 medicines were prescribed which gives an average of 2.19 medicine per patient. The availability of the prescribed medicines in the pharmacies was 1061 (75%), and the affordability of medicines was 1.93 WD that indicates an unaffordable price. The average written medication labels score of 3.1 out of 8 points and the average patient knowledge score for correct usage of medicines was 4.5 out of 6 points. Overall, 585 (90%) of patients reported being satisfied with pharmacy services; the counseling skill of pharmacists 609 (93.7%), and dispensing area 607 (93.4%) cited the most. The only significantly associated factor for satisfaction was the infrastructure of the pharmacy.

**Conclusion:**

Overall satisfaction of patients with the auditable pharmaceutical transactions and services implemented in hospital pharmacy services was generally high. The participants were most satisfied with the pharmacist counseling and dispensary area. The medication availability is moderate but the cost is unaffordable. Advanced infrastructures have resulted in a significant improvement in patient satisfaction.

**Supplementary Information:**

The online version contains supplementary material available at 10.1186/s40545-021-00372-1.

## Introduction

Ensuring the availability and rational use of medicines is a vital component of patient care service that hospital pharmacies should provide. The hospital pharmacy services should be provided in a convenient work environment that facilitates quality workflow and guarantee satisfaction for patients and the staff [1]. Improved communication between pharmacy professionals and patients, convenience, and courtesy of a pharmacy creates client satisfaction and ultimately improved treatment outcomes [2].

Patient satisfaction is an integral component of quality indicator that reflects the feeling of patients towards services provided. It is the congruence between the expectation and actual health care service received from the patient perspective [2–4]. The overall quality of health care and patient satisfaction is dependent on the consistent provision of higher quality pharmacy service. Satisfaction with pharmacy services promotes adherence of patients on medications and achieve desired treatment outcome [3]. Hence, patient satisfaction is a widely used indicator to identify potential areas for improvement [5].

Ensuring the availability of medicines and improving dispensary premises, workflow, and quality pharmaceutical services result in patient satisfaction towards pharmacy services. Besides, reducing waiting time, promoting privacy, and increasing care times are vital for patient satisfaction. Such services create an environment, whereby patients are empowered to make an informed decision about prescribed medicine. Moreover, patients will be protected from unnecessary harm associated with irrational use of medicine [1, 6].

Studies on the rate of patient satisfaction vary among studies as it is a subjective measure. A study in Malaysia found relatively high patient satisfaction with outpatient pharmacy services [5]. However, it is reported that only one-third of patients were satisfied with the outpatient pharmacy service offered in Zambia [7]. The age, education, frequency of visit, self-perceived health status, and general knowledge of pharmacists were factors related to patient satisfaction towards pharmacy service [5].

In Ethiopia, institution-based studies found slightly higher than 50% overall patient satisfaction towards outpatient pharmacy services which is generally low [2, 3, 8–10]. The patients score higher satisfaction with the promptness of prescription medication service [2], and professionalism of the pharmacy staff [3] but lower on the information provided on storage, and possible side effects of medication. Patients who were served free of payment, illiterates, older adults, and those with no job compared showed better satisfaction [2, 10].

Several factors affect patient satisfaction towards pharmacy service. Patient satisfaction is positively influenced by service promptness, pharmacist attitude, medication counseling, pharmacy location, and waiting area [11]. In addition, socio-demographic characteristics (age, gender, marital status, health status, educational level, residency, and ethnicity), patient expectation, pharmacy location, medication availability, and affordability influence satisfaction [7, 12, 13]. The leading factors affecting patient satisfaction were the non-availability of medicines at the dispensary, lack of privacy and empathy from pharmacy professionals, and long waiting times at a pharmacy [7, 14].

Poor structural and system problems such as transparency and accountability; recording, documentation, and information management; human resources, pharmacy workflow in Ethiopia resulted in patient dissatisfaction and reduced trust [15]. To improve the quality of pharmacy services and ultimately increase patient satisfaction, an innovative approach named auditable pharmaceutical transactions and services (APTS) is being implemented in public hospitals in Ethiopia since 2012. APTS requires a change in the system and infrastructure of the pharmacy dispensary that facilitates good workflow. Reports found that the implementation of APTS brought notable improvement in the pharmacy service and patient satisfaction through improving the availability of medicines, upgrading premises, dispensing workflow, and providing quality pharmaceutical services [16]. Nowadays, to alleviate poor pharmaceutical management and improve the overall quality of pharmacy services, the Ethiopian Federal Ministry of Health (FMOH) is expanding the implementation of APTS in all public hospitals. Therefore, this study was aimed to assess patient satisfaction in hospital pharmacies that implemented the APTS system in Ethiopia.

## Methodology

### Study area

This is a national study conducted to assess the impact of the APTS system on the quality of pharmacy service and patient satisfaction. Ethiopia has ten regional states and two city administrations. Currently, the APTS system is implemented in 224 health facilities in Ethiopia. This study was conducted in 26 public hospitals (9 referral hospitals, 14 general hospitals, and 3 primary hospitals) that have implemented the APTS.

### Study design

This study was based on a cross-sectional study design that was conducted from September 5 to October 5, 2020.

### Data collection methods

The data collected was conducted using quantitative methods based on face-to-face patient interviews and checking the provision of written labels for dispensed medication upon leaving the pharmacy.

### Study population

Source population includes all patients served in public hospital pharmacies in all regional states and two city administrations of Ethiopia. The study population were all patients who got pharmacy service in the selected 26 selected hospitals of Ethiopia during the study period. In each hospital, 25 patients were randomly selected.

### Sample size and sampling technique

The involved hospitals were identified using a multistage sampling technique, whereby APTS implemented hospitals were stratified based on the regional states and the city administrations of Ethiopia. Then, based on the geographic convenience and number of APTS implementing hospitals in each stratum the hospitals to be included were selected. Accordingly, 26 hospitals were identified and 25 randomly selected patients in each facility were included in the study that gives 650 total participants.

### Inclusion and exclusion criteria

The patients who received pharmacy service from the selected hospitals and who voluntarily gave verbal consent to participate in the study were included. In addition, caregivers were interviewed for children who cannot give consent. Patients who were unable to provide the required information or unwilling to participate in the study were excluded.

### Data collection techniques

The data were collected using the structured and pilot-tested questionnaire and standard observation checklist. The data abstraction format contained questions on participants' demographic data, availability of prescribed medicines, medication cost, labeling information, and knowledge on the correct use of dispensed medicines. The observation checklist was employed to review the written medication labels provided to the patient at the pharmacy. Two data collectors were assigned in pairs to collect data from two hospitals that are located in their regional states. The data collectors were trained for 1 day on how to approach patients, use the questionnaire to conduct an interview, and record the required data on the data abstraction form. The data collection process was closely followed by the national technical working group organized at the ministry of health, pharmaceutical, and medical equipment directorate. The group monitors the daily progress via telephone, receiving progress reports, and providing direction on each step of data collection.

### Data analysis

Descriptive statistics such as frequencies, proportions, and ratios are used to present the data using an excel spreadsheet. The completeness of the written label information was scored by assigning 1 for the correctly labeled information and 0 for missed label information. The necessarily labeling information according to the good dispensing practice such as availability of patient name, drug name, strength, dose, frequency, quantity/duration, date of dispensing, and precaution were scored. In addition, scoring was performed regarding patient knowledge on correct medication use by assigning 1 for correctly recalled component and 0 for missed information. Six essential components for the correct medication use such as dose of medicine, route of administration, frequency of administration, duration of therapy, storage condition, and precautions were scored. The association between patient satisfactions with the pharmacy services was analyzed using SPSS version 23. Using the average satisfaction as a cut point, the associations of independent pharmacy services to the satisfaction of patients was determined using logistic regression analysis. The *p* value < 0.05 was considered statistically significant.

## Result

### Demographic characteristics of study participants

In this study, a total of 650 participants were included. The majority of the participants were females 355 (55%) and with age above 40 years 239 (37%). Table 1 describes the demographic characteristics of participants.

**Table 1:** The demographic characteristics of Study participants, Ethiopia 2020 (*N* = 650)

Demographic characteristics	Frequency	Percent
Sex		
Male	295	45.4
Female	355	54.6
Age in years		
≤18	235	36.2
19–29	75	11.5
30–39	101	15.5
≥ 40	239	36.8

### The infrastructure of dispensary

In Ethiopia, since the implementation of the APTS system in 2012, the design of APTS Pharmacy has been changed 4 times. Out of the 26 included hospital pharmacies, 2 hospital pharmacies were old designs, 4 hospitals were 4-A design, 9 hospitals were 5-A design, and 11 hospitals have the latest 6A infrastructural design. The description of the design types is presented in Additional file 1: Annex 1.

### Written labeling information for patients

Out of a total of 26 hospital pharmacies assessed, 24 hospital pharmacies provide patients with the written label for dispensed medications and 2 hospitals never write a label. However, the contents of the written label provided to patients were not complete as per the national good dispensing practice [19]. The components of labels commonly written for patients upon leaving the pharmacy were frequency of administration 452 (69.5%) and the dose of medicine 419 (64.5%). The precautions 44 (6.8%), date of dispensing 72 (11.1%), and patient name 142 (21.8%) were the least written labels (Table 2). An average score for label written practice was 3.1 out of 8 components assessed to be labeled.

**Table 2:** The components of written labeling and patient satisfaction with provided services at APTS dispensary pharmacies (*n* = 650)

Components	Frequency	Percent
Provided written label information		
Patient name	142	21.8
Drug name	326	50.2
Strength	330	50.8
Dose	419	64.5
Frequency	452	69.5
Quantity/duration	241	37.1
Date of dispensing	72	11.1
Precautions	44	6.8
Patient satisfied with		
Dispensing area	*607*	*93.4*
Dispensing process	*582*	*89.5*
Counselling skill of pharmacist	*609*	*93.7*
Privacy	*564*	*86.8*
Assistance to the patient	*555*	*85.4*

### Patient knowledge on correct medication use

**Fig. 1:**
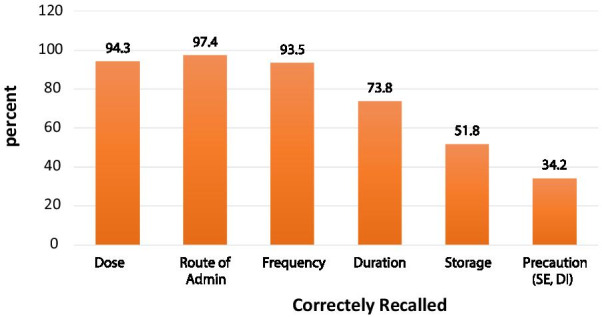
Patient knowledge on the correct dosage for dispensed medicines, Ethiopia, 2020

The knowledge of patients on dispensed medicines was assessed based on the WHO drug use indicators on the patients’ knowledge of correct doses [20]. Accordingly, most respondents correctly recalled route 633 (97.4%), and frequency 613 (94.3%) of administration. However, only 337 (51.8%) and 222 (34.2%) participants recall the correct storage and precaution of the medications they use, respectively (Fig. 1). The score of patient knowledge on correct medication use was found to be 4.5 out of 6 points.

### Availability of medicines

A total of 1422 medicines were prescribed to all 650 study participants during the assessment period, which gives an average of 2.2 drugs per prescription. Out of the prescribed medicines, 1061 (75%) medicines were dispensed to patients in the respective pharmacies of the hospital. The availability of prescribed medicines in the hospital pharmacies ranged from 61% in Afar hospital to 100% in Michew Hospital (Table 3).

**Table 3:** The patient satisfaction and Pharmacy Services in APTS implemented Hospital Pharmacies, Ethiopia, 2020

Name of hospitals	Patient satisfaction (%)	Dispensary design type	Availability (%)	Drugs per Rx	Labeling score/8	Knowledge score /6
Adama Hospital	84	5A	62	2.64	4.36	3.96
Addis Alem HL	51.2	4A	72	1.84	0.16	4.24
Afar 2nd Hospital	98.4	6A	61	2.36	2.56	3.96
Alamata Hospital	90.4	6A	78	2.04	1.28	5.12
Ambo Hospital	98.4	6A	82	2.28	5.2	4.48
Assosa Hospital	97.6	6A	73	2.08	2.44	4.92
Axum Hospital	95.2	6A	70	1.76	2.6	5.36
Batu Hospital	91.2	6A	71	2.52	2.44	3.4
Bishoftu Hospital	96	5A	76	1.68	3.24	5.64
Buta Jira Hospital	82.4	4A	81	2.56	0.88	4.28
Debre Markos Hospital	77.6	Old	69	2.2	0	5.6
Debre Birhan Hospital	100	6A	94	1.92	1.68	5
Dessie Hospital	51.2	Old	76	1.8	1.28	4.64
Dil Chora Hospital	92.8	5A	77	2.44	5.28	4.12
Dubti Hospital	99.2	6A	70	2.44	2.92	4.16
Enjibara Hospital	99.2	6A	70	2.44	3.76	3.8
Gambela Hospital	90.4	6A	86	1.12	7.16	5.2
Jigjiga Hospital	98.4	6A	85	2.08	6.64	3.88
Jugol Hospital	99.2	6A	70	2.0	5.68	3.8
Michew Hospital	93.6	4A	100	1.64	1.44	5.28
Nekemete Hospital	75.2	4A	70	2.24	0	4.16
Tulu Bollo Hospital	85.6	5A	86	2.36	5.44	3.96
Wolench Hospital	96.8	5A	68	2.76	3.28	5.36
Worabe Hospital	94.4	5A	88	1.92	2.8	3.76
Yekatit 12 Hospital	98.4	5A	68	2.48	3.32	3.68
Zewuditu Hospital	96.8	5A	62	3.28	5.2	3.96
Average	90		75	2.2	3.1	4.5

### Affordability of medicines

The affordability of medicines was calculated according to the WHO affordability index [17, 18] that uses the daily wage (DW) of government employees. The Ethiopian government minimum monthly wage for government employees is 961 Ethiopian Birr (ETB). Therefore, the DW will be 961/30 days = 32 ETB. The average price of medications in the assessed hospitals was found to be 1.93 WD indicating unaffordable price as of WHO criteria. Considering the type of hospitals assessed price of medicines varies. The price of medicines was found to be 3WD in specialized hospitals, 1WD in General hospitals, and 0.69 WD in primary hospitals. This indicates that patients cannot afford the costs of medications to be treated at specialized hospitals.

### Patient satisfaction towards pharmacy services

When asked whether satisfied or not with the service from the pharmacies, most of the participants 585 (90%) responded that they were satisfied with the pharmacy service. The services that participants highly satisfied were the counseling skill of pharmacists 609 (93.7%), and dispensing area 607 (93.4%)***.*** The patient satisfaction towards the pharmacy services varied from hospital to hospital with the range of 51.2% to 100% in the assessed hospitals (Table 3).

### Factors associated with patient satisfaction

The association of medicine availability, dispensary infrastructure, knowledge score, and score of written labeling information with patient satisfaction was assessed with logistic regression. The new dispensary APTS designs have significantly improved patient satisfaction as compared with the older designs. The odds of patient satisfaction above the average 90% were 15 (*p* = 0.047) and 25 (*p* = 0.016) times higher among patients served in 5A and 6A designed dispensaries than older designs (old-fashioned and 4A), respectively. Using average values as a cut point, the patient satisfaction was not significantly associated with proportional medicines availability (*p* = 0.49], knowledge score on correct medication usage (*p* = 0.65], and written labeling information score (*p* = 0.35). Table 4 shows the association of patient satisfaction with pharmacy services and pharmacy dispensary design.

**Table 4:** The patient satisfaction with the pharmacy services and pharmacy dispensary design in APTS implemented Hospitals, Ethiopia, 2020

Variable	Average patient satisfaction		Binary logistic regression		
	< 90% satisfied	≥ 90% satisfied	OR	(95% CI)	*p* value
Proportional availability medicine availability					
< 75%	4	10	1		0.49
≥ 75%	5	7	1.786	0.35–9.13	
Patient knowledge score on correct dosage					
< 4.5	5	11	1		0.65
≥ 4.5	4	6	1.47	0.28–7.63	
Labeling medicine score					
< 3	6	8	1		0.35
≥ 3	3	9	2.25	0.42–12.1	
Type of dispensary design^a^					
Old or 4A	5	1	1		0.048
5A	2	6	15.0	1.0–218.3	0.047
6A	2	10	25.0	1.8–346.7	0.016

^a^The types of dispensary design have been improved four times since the start of APTS implementation (old design, 4A, 5A and 6A designs). Detailed description of dispensary designs is included in Additional file 1: Annex 1

## Discussion

This is a national study that assessed the quality of service and patient satisfaction in auditable pharmaceutical transactions and services (APTS) implemented in hospital pharmacies of public hospitals of Ethiopia. According to the principle of good dispensing practice [19], clear and concise medicine information should be provided to patients on the dispensed drugs. One of the quality pharmacy service indicators is providing adequately labeled medications for clients to minimize errors due to forgetting key information counseled at a pharmacy. In this study, 24 hospitals provide written labels to patients for whom medications have been dispended although the completeness of labels varies. The most commonly provided written medication labels were the frequency of administration 69.5% and the dose of medicine 64.5%, whereas the least labeled information were precautions 6.8%, date of dispensing 11%, and patient name 22%. A study in Botswana reported that dosage 77%, name of drug 73%, strength 50%, and the name of the patient 44% were labeled information for dispensed medications [21]. Therefore, the dispensing practice of the pharmacists does not comply with the standards of good dispensing practice.

This study revealed that the average number of drugs per prescription was 2.19, which is slightly higher than the WHO recommendation that is less than 2 drugs per prescription [22]. Besides, an average of 75% of prescribed medications was dispensed to patients at the APTS pharmacy of the same hospital. The availability of prescribed medicines in the hospital pharmacies assessed ranged from 61% Afar hospital to 100% in Michew Hospital. Our finding is in line with the availability of 76% of drugs in a general hospital in Ethiopia [23]; however, it is lower than a national report of 86.4% proportional availability of prescribed medicines to be dispensed in the APTS implemented hospital pharmacies in 2016 [16]. In contrast to our finding the lower proportion of clients were receiving all medications prescribed in the hospitals of Eastern Ethiopia 35% [10] and Dessie public hospital 56% [8]. This variation may be due to the differences in methods of measuring the proportional availability of prescribed. In our study, proportional availability is determined by considering the availability of each prescribed medicine in the dispensary, whereas in later studies, availability was determined by the number of patients who received all prescribed medication in APTS implemented pharmacies.

Our study showed 90% of the overall satisfaction of patients towards the pharmacy services. This is similar to the finding of 94% patient satisfaction with their pharmacy visit in Poland (24) but higher than 33% patient satisfaction in Zambia (7), and 46% in Tanzania (25). This finding is also higher than the national APTS assessment finding of 74.5% of overall patient satisfaction (26) and other institution-based findings in Ethiopia, 55% in Gamo Gofa (23), 52% in Tikur Anbessa Specialized and Gondar University hospitals (3,13), 46% in Eastern Ethiopian Hospitals (10) and 59% in Dessie Hospital (8). The variation in patient satisfaction may be associated with the difference in sociodemographic characteristics and the subjective expectation of participants to pharmacy services.

This study showed, most patients were satisfied with the counseling skill of pharmacists 93.7%, and the dispensing area 93.4%. Similarly, the national assessment reported higher client satisfaction with the pharmacist competency 81% and the assistance from pharmacists 75% (26). In addition, this finding is in line with a study conducted in 13 hospitals of Eastern Ethiopia by Ayele et al. that revealed higher satisfaction with pharmacist counseling [10]. These findings may probably indicate the pharmacists were performing professionally and with good professional skills to provide quality pharmacy services to clients. In addition, the renovation of dispensary infrastructures for APTS pharmacy implementation contributed to higher satisfaction with the dispensary area.

Regarding the correct knowledge of patients on dispensed medications, this study revealed that most patients recalled correctly the route of administration 97.4%, frequency 94.3%, and dose 93.5% of medications but the lesser proportion of patients knew the storage 51.8% and use precautions 34.2% for medications dispensed. Similarly, studies reported a higher level of patient medication knowledge on the route of administration, dosage, and frequency of administration [21, 27, 28].

## Conclusion

The quality of pharmacy services provided was satisfying to patients particularly with the counseling skills of pharmacy professionals, care provided, and design of the pharmacy layout. However, the cost of medicines is unaffordable to patients paying out of pocket. The availability of prescribed medicines at the APTS implemented hospital pharmacies need to be improved in many hospitals. In addition, providing patients with adequately labeled medicine is a requirement, most of the patients received unlabeled or incomplete labeled medicines. Advanced infrastructures have resulted in a significant improvement in patient satisfaction.

## Supplementary Information

**Additional file 1: Annex 1.** The description of the infrastructural designs and Pictures for APTS.

## Data Availability

The data sets supporting the conclusions of the study are available with the corresponding author upon request.

## Abbreviations

APTS: Auditable pharmaceutical transaction system
CI: Confidence interval
ETB: Ethiopian Birr
FMoH: Federal Ministry of Health
OR: Adjusted odds ratio
WD: Wage days
WHO: World Health Organization

## References

[1] FMoH. Pharmacy Service. In: Ethiopian Hospital Services Transformation Guidelines. 2017.

[2] Surur AS, Teni FS, Girmay G, Moges E, Tesfa M (2015). Satisfaction of clients with the services of an outpatient pharmacy at a university hospital in northwestern Ethiopia: a cross-sectional study. BMC Health Serv Res.

[3] Semegn S, Alemkere G (2019). Assessment of client satisfaction with pharmacist services at outpatient pharmacy of Tikur Anbessa Specialized Hospital. PLoS ONE.

[4] Al-Abri R, Al-Balushi A (2014). Patient satisfaction survey as a tool towards quality improvement. Oman Med J.

[5] Ismail A, Nee Y, Id G, Id NA (2020). Factors associated with patient satisfaction towards pharmacy services among out- patients attending public health clinics : questionnaire development and its application. PLoS ONE.

[6] Beyene D, Abuye H, Tilahun G (2020). Effect of auditable pharmaceutical services and transaction system on pharmaceutical service outcomes in public hospitals of SNNPR. Ethiopia Integr Pharm Res Pract.

[7] Kalungia AC, Kamanga T (2016). Patients ’ satisfaction with outpatient pharmacy services at the university teaching hospital and Ndola Central Hospital in Zambia. J Prev Rehabil Med.

[8] Kebede H, Tsehay T, Necho M, Zenebe Y (2021). Patient satisfaction towards outpatient pharmacy services and associated factors at Dessie Town Public Hospitals, South Wollo, North-East. Patient Prefer Adherence.

[9] Eshetie G, Feleke A, Genetu M (2020). Patient satisfaction and associated factors among outpatient health service users at primary hospitals of North Gondar, Northwest Ethiopia, 2016. Adv Public Heal.

[10] Ayele Y, Hawulte B, Feto T, Basker GV, Bacha YD (2020). Assessment of patient satisfaction with pharmacy service and associated factors in public hospitals, Eastern Ethiopia. SAGE Open Med.

[11] Khudair I, Corporation HM, Raza SA (2011). Measuring patients ’ satisfaction with pharmaceutical services at a public hospital in Qatar. Int J Health Care Qual Assur.

[12] Adhikari M, Paudel NR, Mishra SR, Shrestha A, Upadhyaya DP (2021). Patient satisfaction and its socio- demographic correlates in a tertiary public hospital in Nepal : a cross-sectional study. BMC Health Serv Res.

[13] Ayalew MB, Taye K, Asfaw D, Lemma B, Dadi F, Solomon H (2017). Patients’/clients’ expectation toward and satisfaction from pharmacy services. J Res Pharm Pr.

[14] Nigussie S, Edessa D (2018). The extent and reasons for dissatisfaction from outpatients provided with pharmacy services at two public hospitals in eastern. Front Pharmacol.

[15] SIAPS-USAIDS. Transforming Pharmaceutical Services in Ethiopia through Auditable Pharmaceutical Transactions and Services. 2014.

[16] Fenta TG, Gulilat B, Teshome D, Sebsibe F, Assefa T. Outcome of Auditable Pharmaceutical Transactions and Services Implementation : Assessment Report. 2016.

[17] WHO, HAI Global, (2008). Measuring Medicine prices, affordability and price availability, components.

[18] WHO. Medicines in Health Systems : Advancing access, affordability, and appropriate use. Bigdeli M, Peters D, Wangner A, editors. 2014.

[19] FMHACA. Manual for Medicines Good Dispensing Practice. 2nd ed. Addis Ababa, Ethiopia; 2012.

[20] WHO. How to investigate drug use in health facilities. 1993. 1–92 p. http://apps.who.int/medicinedocs/pdf/s2289e/s2289e.pdf

[21] Boonstra E, Lindbaek M, Ngome E, Tshukudu K, Fugelli P (2003). Labelling and patient knowledge of dispensed drugs as quality indicators in primary care in Botswana. Qual Saf Heal Care.

[22] WHO. How to Investigate Drug Use in Health Facilities. 1993. p. 92.

[23] Mensa MS, Wogayehu BT (2019). Availability and affordability of essential medicines and patient satisfaction on pharmacy services: case of two public hospitals in Gamo Zone, Southern Ethiopia. CPQ Med.

[24] Mohammed SA, Hailu AD (2020). Patient–pharmacist interaction in Ethiopia: systematic review of barriers to communication. Patient Prefer Adherence.

[25] Jande M, Liwa A, Kongola G, Justin-Temu M (2013). Assessment of patient satisfaction with pharmaceutical services in hospital pharmacies in Dar es Salaam, Tanzania. East Cent Afr J Pharm Sci.

[26] USAID/SIAPS. Auditable Pharmaceutical Transactions and Services (APTS): Findings of the Baseline Assessment at Federal, Addis Ababa, and Teaching Hospitals. 2014.

[27] Fentie M, Mekonnen T, Tessema M, Yeshaw M (2014). Assessment of patients knowledge to their dispensed medications in pharmacies Assessment of Patients’ Knowledge to Their Dispensed. Int J Pharm Chem Sci.

[28] Wogayehu B, Adinew A, Asfaw M (2020). Knowledge on dispensed medications and its determinants among patients attending outpatient pharmacy at Chencha Primary Level Hospital, Southwest Ethiopia. Integr Pharm Res Pract.

