# Supplementary material for: Assessment of patient satisfaction towards auditable pharmaceutical transactions and services implemented in outpatient hospital pharmacy in Ethiopia
Source: J Pharm Policy Pract. 2021 Oct 19;14:83. doi: 10.1186/s40545-021-00372-1 (PMC8527707; doi:10.1186/s40545-021-00372-1)
Supplement: Supplementary file 1 — Additional file 1: Annex 1. The description of the infrastructural designs and Pictures for APTS. [file 40545_2021_372_MOESM1_ESM.docx]

**Supplementary Material**

**Annex 1: The description of the infrastructural designs and Pictures for APTS**

| **Old designs:** the counter height (1.20 meters) and one door only. Height is not appropriate for all patients and one door is not suitable for entrance and exit. In the figure, only one patient can rest his hand on the counter even though it is not comfortable for him. Others couldn’t arrive at the counter since their arm height is less than 1.20 meters. | **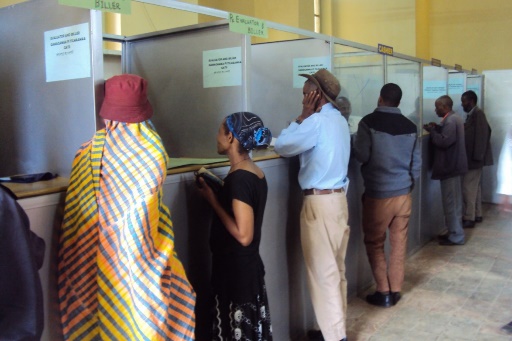** |
| --- | --- |
| **Revised old design:** the counter height (1.15 meters) and one door only. But with closed windows and with narrow holes. The closed window can lead to poor communication  | 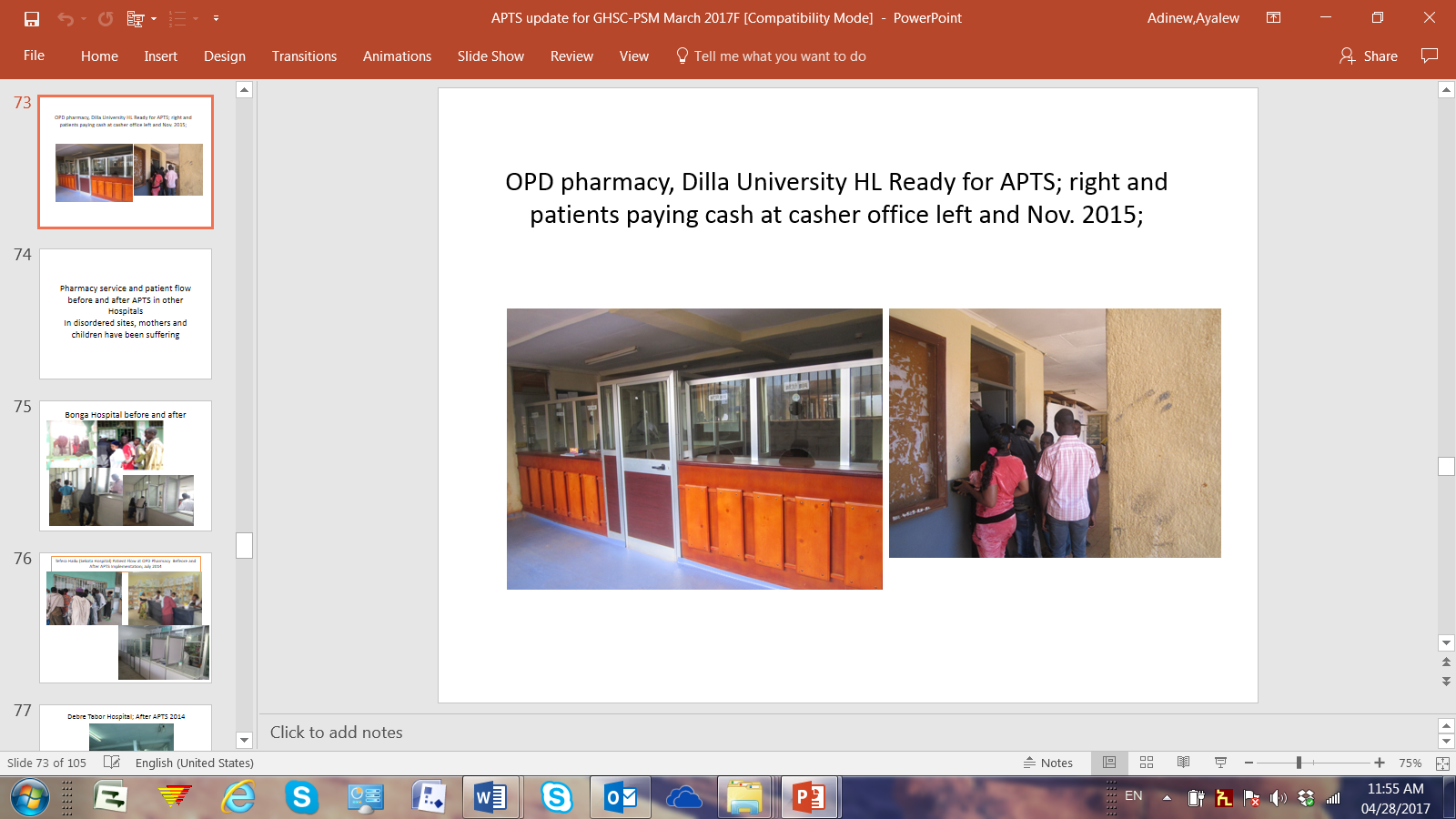 |
| **Model 4-A:** Specification; Height of counters 1.10m. Both pharmacists and patients are standing. This is comfortable for patients and promotes a good communication environment between patients and professionals. However, pharmacy professionals must stand the whole day, no place to put computers and not comfortable for tired patients. | 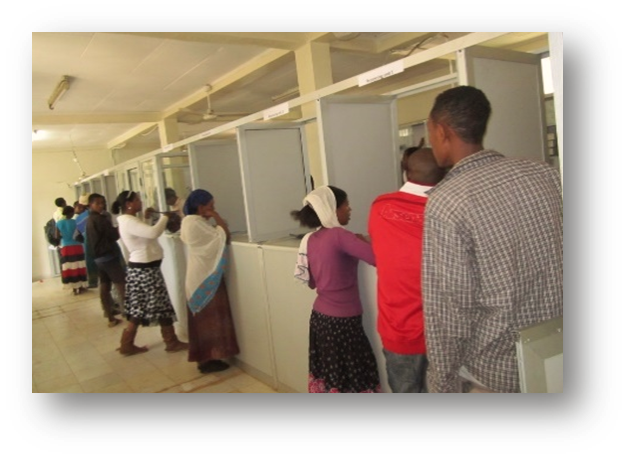 |

| **Model 5-A:** Two counter levels with a height of lower counter 0.75 meters and higher counter with 1.10 meters. Pharmacists can sit, and patients can stand and rest their arms. This allows patients to comfortably rest their arms. The space below the upper counter can be used to put a computer. | 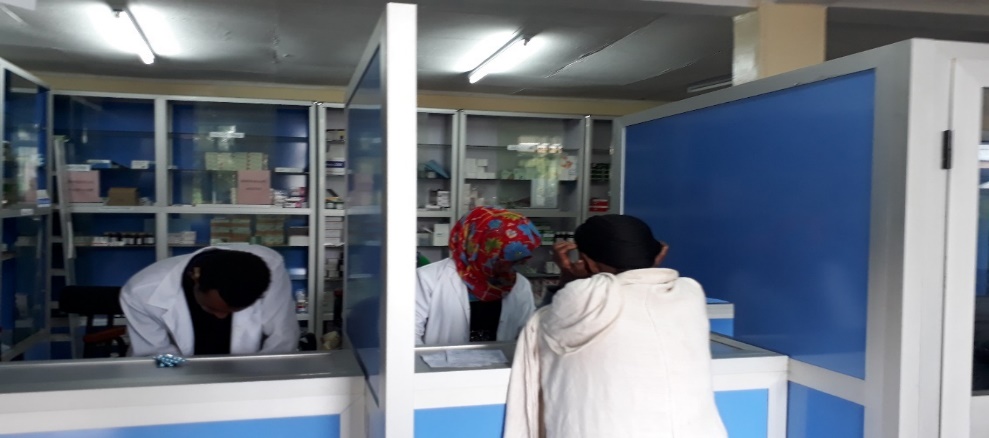 |
| --- | --- |
| **Model 6-A:** Two counters level for computer and with a slanted counter, 1.10 meters in height. Slanted counters to improve patient suitability.  Comfortable for patients because they can rest their arms and will not clash their feet. It has individual counters that professionals can consult, and no one can hear the advice. It has space for other patients to travel to other counters or exit doors too. It has a safe space for computers and is thus suitable to implement electronic APTS. This type of counter increases suitability, safety, efficiency, effectiveness of professionals and is currently recommended to be designed in all health facilities of Ethiopia. It is believed that this type of pharmacy will improve patient and professional satisfaction. | **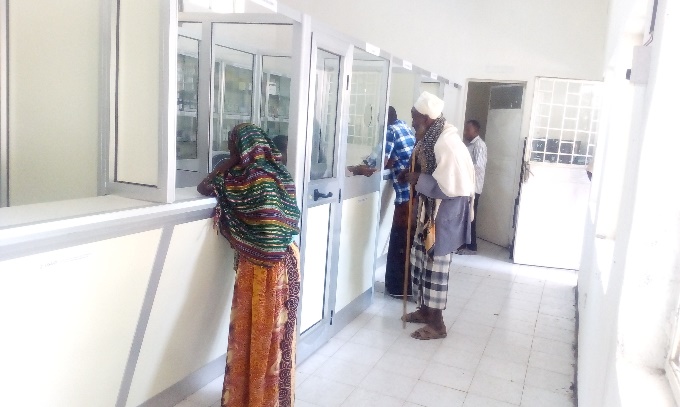** |
